# Development of a Patient-Oriented Intervention to Support Patient-Provider Conversations about Unnecessary Lower Back Pain Imaging

**DOI:** 10.3390/ijerph18052786

**Published:** 2021-03-09

**Authors:** Maryam Madani Larijani, Cindy Dumba, Heather Thiessen, Angie Palen, Tracey Carr, Jason R. Vanstone, Daryl R. Fourney, Collin Hartness, Robert Parker, Gary Groot

**Affiliations:** 1 Community Health and Epidemiology, University of Saskatchewan, Saskatoon, SK S7N 5E5, Canada; maryam.madani@usask.ca (M.M.L.); tlc143@mail.usask.ca (T.C.); 2 Patient and Public Partner of Choosing Wisely Canada, Regina, SK S4S 4V4, Canada; cindydumba@yahoo.ca; 3 Saskatchewan Patient & Family Leadership Council, Saskatoon, SK S7K 7P8, Canada; hrthiessen@gmail.com; 4 Choosing Wisely Saskatchewan, Saskatoon, SK S7K 1P3, Canada; Angie.Palen@saskhealthauthority.ca; 5 Saskatchewan Health Authority, Regina, SK S4P 0W5, Canada; Jason.Vanstone@saskhealthauthority.ca (J.R.V.); Collin.Hartness@saskhealthauthority.ca (C.H.); Robert.Parker@saskhealthauthority.ca (R.P.); 6 Division of Neurosurgery, University of Saskatchewan, Saskatoon, SK S7N 0W8, Canada; daryl.fourney@usask.ca

**Keywords:** lower back pain, patient engagement, patient education, physician-patient relation, Choosing Wisely Canada, diagnostic imaging, primary healthcare

## Abstract

Background: despite the efforts of multiple stakeholders to promote appropriate care throughout the healthcare system, studies show that two out of three lower back pain (LBP) patients expect to receive imaging. We used the Choosing Wisely Canada patient-oriented framework, prioritizing patient engagement, to develop an intervention that addresses lower back pain imaging overuse. Methods: to develop this intervention, we collaborated with a multidisciplinary advisory team, including two patient partners with lower back pain, researchers, clinicians, healthcare administrators, and the Choosing Wisely Canada lead for Saskatchewan. For this qualitative study, data were collected through two advisory team meetings, two individual interviews with lower back pain patient partners, and three focus groups with lower back pain patient participants. A lower back pain prescription pad was developed as an outcome of these consultations. Results: participants reported a lack of interactive and informative communication was a significant barrier to receiving appropriate care. The most cited content information for inclusion in this intervention was treatments known to work, including physical activity, useful equipment, and reliable sources of educational material. Participants also suggested it was important that benefits and risks of imaging were explained on the pad. Three key themes derived from the data were also used to guide development of the intervention: (a) the role of imaging in LBP diagnosis; (b) the impact of the patient-physician relationship on LBP diagnosis and treatment; and (c) the lack of patient awareness of Choosing Wisely Canada and their recommendations. Conclusions: the lower back pain patient-developed prescription pad may help patients and clinicians engage in informed conversations and shared decision making that could support reduce unnecessary lower back pain imaging.

## 1. Introduction

The prevalence of lower back pain (LBP) is substantial in Canada: one in every five Canadians has LBP, and it is the third most common presenting complaint at Canadian emergency departments with about 360,000 annual visits [1,2]. A recent systematic review of appropriateness for LBP diagnostic tests indicated that about one third of imaging requests were considered inappropriate [3]. The misuse and overuse of medical imaging has gained increased national attention in recent years, in part due to the harm and risks associated with radiation exposure from X-rays and computerized tomography (CT) scans, high rates of incidental findings, further tests and surgeries, risk of delayed recovery, and drawbacks related to increasing wait times [3,4,5,6,7,8]. Several national and international organizations have recently highlighted the issue of unnecessary utilization of diagnostic imaging for patients with LBP, including Choosing Wisely Canada (CWC) [9].

CWC recommends performing lumbar spine imaging to confirm the presence of a suspected serious pathology such as an infection, tumor, fracture, cauda equina syndrome, or a neurological deficit (known as red flags) [10]. According to CWC recommendations, imaging is also considered for those patients who are not presenting with above pathological cause but have had up to 6 weeks of medical management and physical therapy with little or no improvement in their LBP [11]. In spite of the availability of clinical practice guidelines to improve the appropriateness of LBP imaging, the prevalence of inappropriate LBP imaging remains high [12,13] and there is ample evidence suggesting that engaging patients and building wider public awareness of unnecessary imaging has been challenging [14,15,16]. CWC discussions about the harms of unnecessary tests tend to not resonate with patients, and studies show that two out of three patients expect to receive imaging for their LBP [16,17,18].

To mitigate the problem of unnecessary imaging, patient education materials have been developed to support patient decisions about not receiving imaging when it is not recommended [19]. Research suggests that informed decision making not only empowers patients by improving their knowledge about treatment and management plans, but also improves satisfaction with the clinical encounter [20]. Several studies show that adoption of informed decision making has the potential to improve patient care; patients who participate in decision making compared with patients not exposed to decision aids, choose less invasive surgical options and more conservative treatment [21].

Despite the importance of the patient role, decision support interventions aimed at reducing unnecessary imaging have traditionally been directed toward healthcare providers, specifically clinicians [22,23,24,25]. One of the main forces that drives overuse may be that patients are not receptive to the message of CWC about risks of over-imaging [26]. Patient expectations and risk of patient dissatisfaction have been indicated as important barriers to reducing overutilization of lumbar spine imaging [26,27]. The focus on the role and impact of patients as key stakeholders in addressing imaging overuse has prompted changes in the CWC framework to engage patients’ values and preferences [28].

Although there is an increasingly growing body of literature pertaining to patient engagement in CWC initiatives, there are a comparatively smaller number of studies that have explored patient perception in the development of patient decision support interventions [29,30,31,32,33,34]. This study explored LBP patient perceptions, employing CWC patient-engagement strategies, in response to the need to develop patient-oriented educational tools to support better patient-clinician communication to avoid inappropriate imaging. This study was developed and conducted in a collaboration with an advisory research team, which included patient partners, researchers, clinicians, healthcare administrators, and the CWC lead for Saskatchewan. We engaged with LBP patients to develop a knowledge translation intervention (e.g., a LBP prescription pad) with the goal of facilitating patient-physician conversations about unnecessary LBP imaging.

## 2. Methods

The study adopted a qualitative research design to develop an understanding of patient-identified key themes in designing a LBP imaging educational intervention. The descriptive nature of qualitative approach allowed for exploring patient perception, alongside the existing literature about LBP knowledge translation interventions. The study explored patients’ perspectives and views through two advisory team meetings, two individual interviews, and three focus group discussions. These methods resulted in the development of the educational patient-oriented LBP prescription pad that helps with physician-patient conversations around unnecessary lumbar spine imaging.

### 2.1. Patient-Oriented Framework

For this study, we employed the CWC patient-developed framework proposed by Born et al., which prioritizes the impact of patient engagement in addressing imaging overuse for LBP [35]. This framework includes four elements that are outlined in Table 1.

### 2.2. Data Collection and Analysis

A multidisciplinary advisory team, which included two patient partners with LBP collaborated to design and conduct this qualitative study to develop the knowledge translation intervention and to seek *why* and *how* the intervention would be successful [36]. The two patient partners were recruited through the Saskatchewan Center for Patient Oriented Research (SCPOR) patient engagement platform. The patient partner, CD, is a patient and public partner of CWC and Choosing Wisely Saskatchewan (CWSK), and the Saskatchewan Appropriateness of Care initiative. Alongside these initiatives, CD has collaborated with the Stop the Line, Regional Infection Prevention and Control (RIPAC), Patient and Family Centred Care Planning Committee, Provincial Surgical Oversight Team, Antimicrobial Stewardship Council, and Senior Leadership Quality and Safety Committee. The patient partner, HT, a co-chair of the Saskatchewan Health Authority Provincial Patient and Family Care Advisory Council and patient partner leader in the Health Standards Organization (HSO), has partnered with the SHA Accreditation Oversight Committee, HSO Technical Committee, Interprofessional Education Curriculum Committee with the Health Sciences Departments at the University of Saskatchewan, and Canadian Foundation for Healthcare Improvement Initiative.

**Figure 1:**
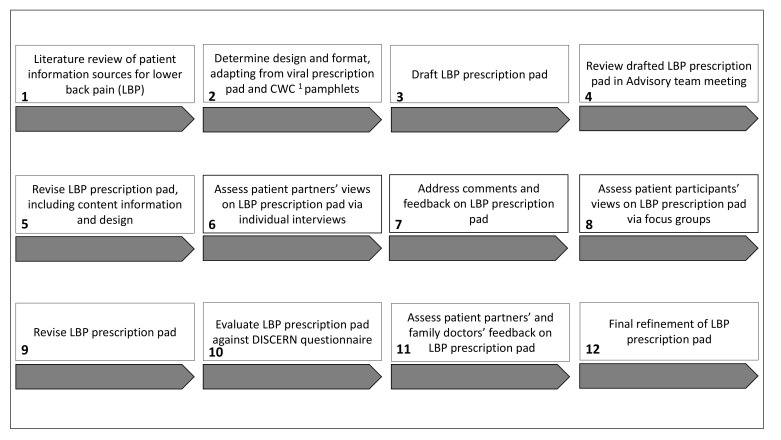
Development process for the LBP prescription pad. ^1^ Choosing Wisely Canada.

In order to develop the LBP prescription pad, stakeholders participated in two 90-min advisory team meetings via a virtual meeting platform (WebEx) and provided input on study design, data collection and methodology, and analysis of results (Figure 1). We also conducted two virtual, in-depth, semi-structured interviews with patient partners, as well as three 90-min online focus groups with patient participants. A patient partner and researcher (M.M.L.) co-led each focus group. Participants were compensated for their time.

Focus group participants were recruited through the University of Saskatchewan website and the SCPOR and SHA patient engagement platforms: a recruitment announcement was posted for a month to invite LBP patients of 18 years of age and older who previously sought care regarding their back problem. In this recruitment, purposive sampling was employed to ensure participants were chosen based on the eligibility criteria [36]. Focus group participant demographics were as follows: nine participants (eight female and one male), participant age was equally distributed between three age groups (three each, aged 18–34, 35–44, and 45–64), and participant occupations included students, nurses, researchers, shop owners, civil employees, and retired/unemployed individuals. Although all participants reported they had LBP for more than ten years, four out of nine received a diagnosis within the past two to five years.

During interviews and focus groups, patient partners and participants were asked about their experiences with LBP and any imaging tests they had received, their knowledge of the benefits and risks of imaging, their experiences with patient-physician conversations about receiving imaging (or not), their knowledge of CWC initiatives and patient resources for LBP, their feedback on the LBP prescription pad (regarding its design and content), and recommendations to improve the LBP prescription pad. Interviews and focus groups were video recorded using the virtual platform software (WebEx) and audio was transcribed verbatim by the Canadian Hub for Applied and Social Research at the University of Saskatchewan. Transcripts were analyzed using NVivo 12 software (produced by QSR International, Melbourne, Australia), reviewed, and coded by two individual researchers (M.M.L. and T.C.).

Key themes were identified through thematic analysis of patient recommendations about what to include in the LBP prescription pad, and themes were compared until consensus was reached between two researchers (M.M.L. and T.C.) [37]. As the focus group data were analyzed, it was determined that we had reached saturation as there were no new themes emerging [38]. By reviewing the researchers’ summary of findings, the patient partners confirmed the validity of the results (codes and key themes) from the focus groups and verified whether the interview results were an accurate reflection of their experiences (member checking) [39]. Using the final themes, the LBP prescription pad was revised by a researcher (M.M.L.) to present LBP patient-tailored information on treatment options, reasons for imaging, risks of unnecessary imaging, and reasons for follow-up. The patient partners actively collaborated in refining the language and content of information of the LBP prescription pad to ensure findings were communicated in an understandable and usable way.

### 2.3. Intervention

**Figure 2:**
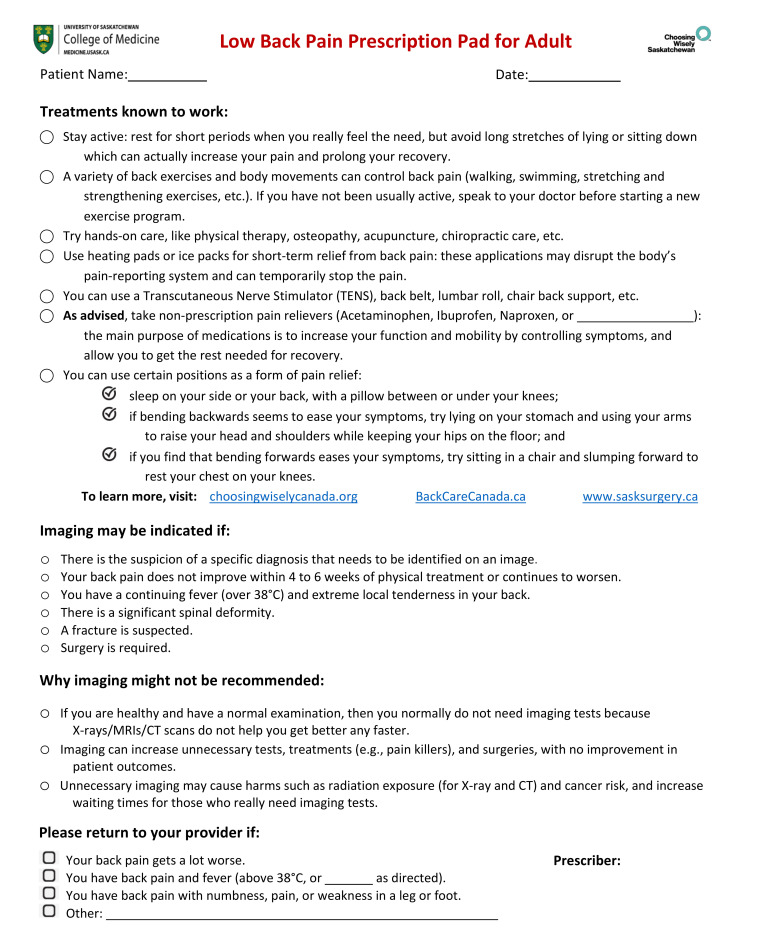
The patient-developed LBP prescription pad.

The content for the “LBP prescription pad” was selected from CWC, BackCare Canada, and Saskatchewan Surgical Initiatives websites (Figure 2). The format and design were adapted from examples of the provincial viral prescription pad and CWC information pamphlets [40,41]. After the first LBP prescription pad was drafted by a researcher (MML), it was presented to the advisory team and revised based on their feedback. Further revisions occurred following consultations with patient partners and these were tested in three patient focus groups. The final design was then evaluated against the DISCERN questionnaire by two researchers (MML and TC) [42]. The DISCERN questionnaire is specifically designed to judge the quality of written health information to promote patient participation in treatment decision-making [42]. The final refinement was carried out by patient partners and physicians who were recruited for the trial, as the intent was to create a prescription pad with greater appeal to both clinicians and patients (e.g., font type, white space, shorter sentences that are more personalized to patients, etc.).

## 3. Results

All participants agreed that developing a LBP prescription pad to promote patient-physician conversations should incorporate patient-identified priorities.

### 3.1. Patient-Identified Content and Format for LBP Prescription Pad

Data from the interviews and focus groups indicated that *Treatments Known to Work*, including physical activity, useful equipment, medications, and additional sources of educational materials, were the most cited content for inclusion in the LBP prescription pad. Participants frequently suggested that it was important that the *reasons for* and *risks of imaging* were both explained on the prescription pad. They all recommended *Treatments Known to Work* should be the foremost information on the LBP prescription pad to show that patients are receiving appropriate care. One participant described their reasoning to begin the LBP prescription pad with treatment options:

*“My doctor didn’t necessarily give me that information, so that’s super handy to have that on the prescription pad so that it’s a talking-point for the doctor to check about that stuff. So, that was super helpful.”*

(Participant 7)

### 3.2. Three Key Themes

In addition to comments and feedback about the content and format of the LBP prescription pad, key themes emerged from data relating to LBP patients’ perceived information about imaging, experience in communication with physicians, and knowledge of CWC. The three key themes retrieved from the focus groups and interviews included: (a) the role of imaging in LBP diagnosis, (b) the impact of the patient-physician relationship on LBP diagnosis and treatment, and (c) the lack of patient awareness of CWC and their recommendations.

#### 3.2.1. The Role of Imaging in the LBP Diagnosis

Patients mostly (7 out of 9) thought imaging is required to diagnose LBP. A patient partner said: “*imaging was [important] to diagnose my seven compression fractures in my back*” (Patient partner 2). Most participants asserted their belief that having imaging was important for treatment of LBP, and they indicated that imaging freed them from the stress of not knowing what was wrong. One participant said:

*“Imaging was important to see if there were any breaks or anything. I was quite athletic and in a lot of sports, so they wanted just to make sure I hadn’t injured myself.”*

(Participant 2)

The other participant also added, “*imaging was important and I wasn’t concerned at all about the risks of the X-ray because I wanted something to happen with my pain*” (Participant 5). Only two participants acknowledged that imaging should not be the first step in treatment: “*Imaging, I think, should be the last thing on your list*” (Participant 8); “*I think we should […] try physio and massage first, […] see if that can work on you first before we start doing invasive testing like CT scans and everything else*” (Participant 4).

In all cases, patient participants reported that their imaging referrals were requested by family physicians or specialists, except one case in a rural area where imaging (X-ray) was ordered by a physiotherapist. In most instances, an imaging test was not ordered prior to initiation of various therapeutic modalities (e.g., massage therapy, acupuncture, aqua-fit, physiotherapy, chiropractic services, etc.). X-rays or CT scans were reported as the first diagnostic imaging test ordered for LBP patients.

In some cases, participants felt that imaging was ordered only because their doctor was rushed and only had time for a very short consultation. It was suggested that a thorough examination of a patients could prevent unnecessary imaging: “*I learned that my three major imaging tests were all unnecessary because you don’t need imaging tests to diagnose [my syndrome], you just need to examine the patient*” (Participant 3). On the other hand, two LBP patients who have had multiple imaging tests thought the advantages of imaging definitely outweighed the disadvantages:

*“I went for an X-ray, where this time, I asked my doctor to give it to me because […] I wanted to see what was going on because it seemed to have gotten worse with the nerve pain down my leg.”*

(Participant 4)

Similarly, one patient participant stated:

*“I thought it was just obviously necessary because I had been in a car accident and then I was having back pain. But I think the second time that I had [imaging] done, it just kept coming back and getting worse, and depending on what I was doing, I felt I should have another [imaging test] just to make sure there wasn’t something wrong. But I haven’t had another [imaging test] since then.”*

(Participant 7)

In terms of the harms and risks of imaging, participants only talked about the exposure to radiation for X-rays and CTs and asserted that magnetic resonance imaging (MRIs) are much more harmful than X-rays and CTs.

#### 3.2.2. The Impact of the Patient-Physician Relationship on the LBP Diagnosis and Treatment

Four patients’ experiences showed a lack of interactive and informative communication was a significant barrier to receiving appropriate care for LBP. Six out of nine patients mentioned a lack of clarity about “what happens next”. They expressed frustration with follow-ups and experiencing many visits or seeing multiple doctors before receiving a diagnosis and treatment plan. To tackle this, a patient partner suggested the need for a culture change in the sense that patients should not feel guilty about asking questions and/or saying too much:

*“We don’t let financial advisors mess with our finances, our mechanics mess with our cars, our veterinarians mess with our pets, unless we know exactly what’s going on. We have to be that vigilant with our own healthcare.”*

(Patient partner 1)

Focus group data showed that patients who have a consistent primary care physician, and especially those who had good and trusting relationships with their physician, were much more satisfied in getting a diagnosis and coming up with a treatment plan. One patient partner asserted that,

*“Even though [patients] all have lower back pain and have been through similar journeys as far as testing, treatments, etc., the experience for those who have trusting open relationships with their physicians is much more positive.”*

(Patient partner 1)

The other patient partner agreed,

*“[I]t is about having conversations between physicians and patients and respecting the lived-experience voice that we bring of living with our condition and all we bring, it has to be respected. And this happens when you have great relationships […] and I know my family doctor does the same thing.”*

(Patient partner 2)

#### 3.2.3. Lack of Patient Awareness of CWC

Our interviews and focus groups indicated none of the participants were familiar with CWC: “*[w]hen I went through [the LBP prescription pad], I went and checked out those websites. I’d never heard of CWC”* (Participant 8); “*I’ve seen probably 20 doctors since 2014 and I’ve never heard of this*” (Participant 2). However, participants all showed interest in learning more about CWC, asking questions about the organization’s goals and recommendations. Participants also emphasized the critical role of patients in promoting appropriateness of LBP imaging:

*“That’s the challenge, Choosing Wisely, is to move the initiative and get it rolling so that more people know about it. That’s our challenge. There are more and more physicians practicing wisely, so it’s coming, but it’s slow. So, [patients] can be a great help. All of [us].”*

(Participant 6)

In general, patient participants were very supportive of the LBP prescription pad, and according to one patient partner: “*it’s clear that [patient participants] believe more informed patients are happier patients*” (C.D.). Almost all participants (8 out 9) emphasized the importance of the LBP prescription pad not being used only as a handout, but to be used for promoting better physician-patient communication: “*if I received this from a doctor who was having a conversation with me like they should, then I think it would be really useful and helpful*”, however,

*“If I went to the walk-in clinic, which a lot of people don’t have family doctors unfortunately, and a walk-in doctor said: we’re not going to do anything and handed this to me, I think I would feel kind of like he was writing me off.”*

(Participant 6)

Similarly, one patient partner said: “*hopefully the physician will invest enough time into having the conversation with the patients rather than just handing them out as paper and making more confusion*” (C.D.).

## 4. Discussion

Effective communication between health care providers and patients is central to health literacy and shared decision-making [43]. For this study, a patient-oriented framework was employed to include patient perceptions in the development of certain CWC education materials that have not been extensively explored in the literature. Using this framework, we ensured that the LBP prescription pad we developed contains information identified as important by patients and meets their needs in promoting conversations with primary care physicians about LBP management. Along with participating in developing a patient-centered knowledge translation tool, patients had an opportunity to increase their knowledge about CWC and were encouraged to participate more fully in their own healthcare decision making regarding LBP testing, diagnosis, and management.

The results indicate that patients support the development of a LBP prescription pad which includes information about treatments options, reasons for why imaging may or may not be required, and why to seek follow-up care. Participants suggested exercise therapy, message and physical therapy, lumbar support and Transcutaneous Electrical Nerve Stimulation (TENS) as effective treatment options for LBP. A systematic review of nonpharmacologic therapies for LBP by Chou et al. indicated exercise therapy is moderately effective for LBP, compared to other therapies such as acupuncture, lumbar support and TENS where there is fair evidence of efficacy for treating LBP [44]. The novel design of the LBP prescription pad with accurate and easy-to-understand information has the potential to shift patient expectations and promote conversations about appropriate imaging and imaging-related risks. Similar studies with patient-focused educational interventions have also shown their significant impact on the facilitation of patient-centered discussions about imaging and increasing their understanding of the harms of unnecessary imaging [45,46].

Our results suggest that patients will benefit most from the LBP prescription pad, if this document is employed as an educational tool to promote a conversation about the treatment plan during a physician consultation and not simply used as a handout. This result is in line with previous research studies [47,48,49]. A study on a similar tool used for antimicrobial stewardship also showed patients’ preference to receive information from a viral prescription pad both verbally and in written format [40].

Our findings also indicate a lack of patient awareness of CWC initiatives, in spite of the efforts of both national and provincial campaigns. This indicates the need to prioritize patient engagement when developing such interventions and other clinical decision support tools. Research has highlighted the role of patient engagement in motivating patients to participate in their own healthcare decisions [50]. Findings from our study contribute to the knowledge of the importance of partnering with patients to increase their capacity, for meaningful, active participation in all aspects of the research.

Data on the role of imaging in the LBP diagnosis demonstrate that, although all participants understood the value of CWC recommendations in terms of reducing low-value care, they mostly believed all of their own imaging tests (more than one for some participants) were performed appropriately. In other words, patients expected their doctors to order imaging prior to other investigations for a timely diagnosis and accepted their doctor’s decision to prescribe imaging to identify the nature of the problem. These findings align with previous research studies indicating that patients overestimate the benefits and underestimate the harms of low-value tests [7,18]. Another barrier that hinders the adoption of evidence-based guidelines and interventions can be physicians’ contradictory desires to both follow guidelines and have a personalized approach for each patient: “my patient is different” [51,52].

The focus group discussions about risks of over imaging reveal patient confusion around the harms of different types of LBP imaging (X-ray, CT, MRI). It was declared that MRI is far more harmful than X-rays and CTs. However, exposure to radiation was only discussed as a risk of over-imaging including X-ray, CT, and MRI. Including basic educational information about harms of imaging overuse (other than ionizing radiation for X-ray and CT) on the LBP prescription pad and supplemental discussions with patients about the risk and harms could be an effective strategy for reinforcing or expanding patient education of the disadvantages of unnecessary, repetitive, and routine lumbar spine imaging and improving appropriateness of LBP imaging.

Patient discussions indicate a good patient-doctor relationship promotes shared decision-making and better communication about imaging overutilization. However, patients who visited their family doctors did not feel the urge to question their decision to order imaging and affirmed that their doctor made the right decision by requesting imaging as the quickest assessment of their illness. Similarly, these patients did not discuss about the risks and harms of unnecessary imaging with their family doctor as it was not perceived to be necessary. An Australian study about patients’ perceptions of a CW patient information tool also indicated patients’ preferences not to question their doctors, given “they trusted their doctor and their doctor’s knowledge, and complied with their doctor’s suggestions and decisions” [53] (p. 4).

All the key themes obtained from patient interviews and focus groups discussions were incorporated along with the patient- identified content and format as a guide in the development of the LBP prescription pad. They also informed the implementation of the intervention in the next phase of this study.

### 4.1. Future Research

Future work includes the trial of the LBP prescription pad with LBP patients and healthcare providers to evaluate its impact in supporting patient-physician conversations and avoiding unnecessary imaging. Since the development of the LBP prescription pad was completed, we have invited healthcare providers, including family physicians and chiropractors, to trial the LBP pad in our province. The results will deepen our understanding of patient and physician perceptions of the LBP prescription pad and will link evidence to practice in terms of promoting effective conversations and supporting successful implementation of CWC recommendations. The findings will lay the groundwork for developing patient-oriented education materials for other CWC recommendations and provide the details of partnership with patients in the province, nationally, and internationally.

### 4.2. Limitations

This study did not collect the demographic data on health literacy; therefore, it is unclear whether the patients who consented to the study had a different level of health literacy from other populations, and if their ability to comprehend health information was different. We recruited patients from various patient engagement platforms including SHA, SCPOR, and university website to ensure the sample represented a heterogeneous group of patients with various levels of health literacy. However, research studies about patient participation in decision making demonstrated that not all patients want to participate in research and more educated people are more likely to volunteer as patient advocates [54]. In the trial of the LBP prescription pad throughout the province, health literacy of patients will be assessed in an online questionnaire designed to seek their feedback after the use of this intervention in their visits for LBP. The number of female participants who volunteered to participate in this study was far greater than the male participants, given there was no screening requirements regarding gender. Studies indicated that women are more likely than men to engage in clinical research about decision making [54]. The higher rate of LBP prevalence in women could be another reason for this difference [55]. The trial will explore the perceptions of more LBP patients from across Saskatchewan to assure the LBP prescription pad will be a helpful knowledge translation intervention for all LBP adult patients regardless of gender. Considering the interviewer was the intervention developer, the engagement of patient partners and the advisory team mitigated potential bias on the part of the researcher and increased the face validity of the LBP prescription pad.

The study was designed to develop a knowledge translation intervention that promotes patient-provider communication to avoid unnecessary imaging. However, we were unable to assess the impact of the intervention and whether the use of the tool resulted in any changes in the patient perceptions and expectations associated with imaging tests. Future studies are planned to address this patient-level outcome.

## 5. Conclusions

Our patient-oriented LBP prescription pad provides patients with information on the risks and benefits of imaging and facilitates discussions between patients and their doctors. This tool may empower active patient participation in medical decisions while also supporting physicians to address patient concerns and uncertainty about their condition. In addition to advancing the clinician-patient relationship, the implementation of the LBP prescription pad has the potential to reduce inappropriate imaging use while educating patients about risks of unnecessary imaging.

## Figures and Tables

**Table 1:** Choosing Wisely Canada Patient-Oriented Framework.

Level of Engagement	Description	This Study
Partner	Establish partnership with patients	We partnered with two patient partners with LBP ^1^.
Engage	Engage patients in key role of planning, steering, and implementing.	The two patient partners were engaged in each step of the process, proposing the research questions, designing participant recruitment materials, data collection, analysis and interpretation of results, and refining the LBP prescription pad.
Inform	Inform patients of benefits, harms and uncertainties about tests, treatments and other procedures.	Exploring views and perspectives of patient partners and participants, we developed a LBP prescription pad as a knowledge translation intervention about treatment options, reasons for imaging, risks of unnecessary imaging, and follow-up to manage LBP.
Empower	Empower patients for shared decision making with healthcare providers.	The LBP prescription pad is intended to promote better patient-clinician conversations about unnecessary imaging.

^1^ Lower back pain.

## Data Availability

The data presented in this study are available on request from the corresponding author. The data are not publicly available due to restrictions regarding the Ethical Committee Institution.
